# Gene expression profiling of CD4^+^ T cells in treatment-naive HIV, HCV mono- or co-infected Chinese

**DOI:** 10.1186/1743-422X-11-27

**Published:** 2014-02-13

**Authors:** Lina Yi, Jin Zhao, Jing Lu, Ying Chen, Lin Chen, Jinquan Cheng, Yan Sun, Zhi Li, Ruotin Men, Li Yang, Hsiangfu Kung, Zhengrong Yang, Ming-liang He

**Affiliations:** 1 Stanley Ho Center for Emerging Infectious Diseases, and Li Ka Shing Institute of Health Sciences, Faculty of Medicine, The Chinese University of Hong Kong, Hong Kong, China; 2 Shenzhen Center for Disease Control and Prevention, Shenzhen, China; 3 Guangdong Provincial Institution of Public Health, Guangdong Provincial Center for Diseases Control and Prevention, Guangzhou, China; 4 College of Life Science, Shanxi Normal University, Xi’an, China; 5 Department of Gastroenterology, Huaxi Medical College, Sichuan University, Sichuan, China; 6 Prince Wales Hospital, Faculty of Medicine, The Chinese University of Hong Kong, Li Ka Shing Medical Science Bldg, Hong Kong, China

**Keywords:** HIV, HCV, Co-infection, Microarray, CD4^+^ T cells

## Abstract

**Background:**

Because of the shared transmission routes, co-infection with human immunodeficiency virus (HIV) and hepatitis C virus (HIV) is very common. Accumulated clinical evidence showed that one could alter the infectious course of the other virus in HIV and HCV co-infected individuals. However, little is known on the molecular basis of HIV/HCV interactions and their modulations on hosts.

**Methods:**

In this study, treatment-naive HIV, HCV mono-/co-infected individuals with CD4^+^ T cell counts >300/μl were recruited and their gene expression profiles were investigated by microarray assays. The differentially expressed genes were identified and validated by quantitative real-time PCR (qRT-PCR). To further understand the biological meanings of the gene expression profiles in these three groups, GSEA analysis (version 2.0, Broad Institute http://www.broad.mit.edu/gsea) was performed.

**Results:**

By gene set enrichment analysis, we revealed that gene sets of cell cycle progression, innate immune response and some transcription factors in CD4^+^ T cells were mainly affected by HIV; while genes associated with GPCR signaling were the major targets of HCV. Metabolic pathways were modulated by both HCV and HIV viruses.

**Conclusions:**

This study for the first time offers gene profiling basis for HCV/HIV mono-/co- infections in human beings. HIV infection displayed the great impact on transcription profile of CD4^+^ T cells in HIV/HCV co-infected individuals. Genes related to cell cycle arrest were significantly mediated by HIV which may lead to dysfunction of CD4^+^ T cells and acceleration of HCV-related disease progression in the co-infections.

## Background

Co-infection with human immunodeficiency virus (HIV) and hepatitis C virus (HCV) is very common, presumably due to their shared transmission routes (*e.g*., drug injection, sex behaviour) [1,2]. It is estimated that over 5 million people are HCV/HIV co-infected worldwide [2]. The prevalence of HCV/HIV co-infection varies widely in different risk groups, with an especially high rate among HIV-positive intravenous drug users (IDUs) [3]. In the United States and China, 72% to 90% IDUs in HIV-infected persons were co-infected with HCV [4].

Accumulated evidence showed that one could alter the course of infection of the other virus in HIV and HCV co-infected individuals. On the one hand, a higher rate of HCV viral persistence and increased viral load are more common in the HCV/HIV co-infected patients than in HCV mono-infected ones [5]. Moreover, it was suggested that HIV could accelerate the progression of HCV-associated liver diseases, including fibrosis, cirrhosis and end-stage liver disease [6-8]. On the other hand, an acute HCV infection normally raises the HIV-induced viremia in persons with otherwise well-controlled illness, and accelerates the progression to AIDS and death by impairing immune reconstitution [9]. Besides, the risk of hepatotoxicity from highly active antiretroviral treatment (HAART) may be increased in the co-infected patients as compared to the HIV mono-infected patients, thus lead to a decreased tolerability for the anti-HIV treatment [10].

Mechanisms of the accelerated progression of diseases in the co-infected patients are not yet well understood. Analysis of gene expression profiles in the HCV/HIV mono-infected or co-infected patients would provide a unique opportunity to understand the mechanisms. It is reported that factors, such as direct viral effects, the immunologic alterations especially the specific T-cell response, have played important roles in the disease progression [11-13]. CD4^+^ T cells, the major HIV target cells, also play an essential role in HCV clearance [14,15]. In this study, we performed the microarray studies to analysis the gene expression profiles of CD4^+^ T cells from treatment-naïve HIV/HCV mono- and co-infected individuals. Moreover, via gene set enrichment analysis (GSEA), a network of enriched pathways related to the pathogenesis of disease progression in the co-infected patients was identified. To our knowledge, this is the first study to analyze the impact of HIV/HCV co-infection at gene transcription level, and our data may offer new insight into understanding the interplay during HIV and HCV co-infection.

## Results

### Differentially expressed genes

Microarray analysis was performed to identify the altered transcripts in 3 study groups with HIV, HCV mono-infection and HCV/HIV co-infections, and the raw data has been deposited in the ArrayExpress database (access number: E-MEXP-3601). Pairwise comparisons from the three study groups (HCV-mono versus HIV-mono, HCV/HIV co-infected versus HCV-mono, HCV/HIV co-infected versus HIV-mono) were carried out. Differentially expressed (DE) transcript identifiers (DETIs) with >2 fold change and p < 0.05 were identified for each comparison. The number of DETIs identified in each comparison is listed in Table 1. The detailed information of each DETI in each comparison was listed in Additional file 1: Table S2.

**Table 1:** Number of differentially expressed transcript identifiers (fold change > 2 and P < 0.05) and enriched gene sets (FDR < 0.01) in pair wise comparisons for CD4+ T cells

	**HCV-mono**		**HCV/HIV co-infected versus**		**HCV/HIV co-infected versus**	
	**versus**					
	**HIV-mono**		**HCV-mono**		**HIV-mono**	
	**up**	**down**	**up**	**down**	**up**	**down**
**Differentially expressed transcript identifiers**	19	35	109	79	44	7
**Enriched gene sets**	6	44	9	10	0	2

To identify the important categories from the DE genes, functions of each DE gene were inspected. Of 54 DE genes identified in comparison between HCV and HIV mono-infected groups, 24 showed significant matches with the known genes. Functional analysis revealed that genes involved in immune system development, immune response (CXCL10, OAS3, CD38, SERPING1, CCR1, FCGR3A and IFI44L) and G-protein coupled receptor (GPCR) signaling pathway (P2RY13, GPR56, CX3CR1 and FFAR2) were differently affected by these two viruses. In the case of HCV/HIV co-infection versus HCV mono-infection, 72 genes with known function were identified. Many of them (16/72) played important roles in stimulus response and immune system process, including: RSAD2, IL6, C1QC, FCGR1C, LILRA3, IGSF6, IL1RN, OAS3. Besides, genes involved in regulating locomotory behavior such as CCRL2, CX3CR1, FPR2 and IL8RA were also shown different expression profiles between these two groups. In comparison between the HCV/HIV co-infections and the HIV mono-infections, only 7 were identified to be known genes. 3 were small nucleolar RNAs, and the remaining 4 were TNFAIP6, AQP9, IL6 and PTX3. The later four genes play important role in immune response and involve in many human diseases, such as cancers and diseases caused by a variety of virus infections.

### Gene set enrichment analysis

To unravel the biological mechanisms differentiating between the HCV/HIV mono- and the co-infected groups, pairwise comparisons using GSEA were performed for all the three groups (HCV-mono versus HIV-mono, HCV/HIV co-infected versus HCV-mono, HCV/HIV co-infected versus HIV-mono). Rather than single DE genes, GSEA evaluates microarray data at the biological pathway level by performing unbiased global searches for genes that are coordinately regulated in predefined gene sets. The number of significantly enriched gene sets (FDR < 0.01) in each pairwise comparison is listed in Table 1. And pathways identified in gene set enrichment analysis (GSEA, FDR < 0.05) were shown in Additional file 2: (Table S3).

### Global gene expression profiles between HIV mono- and HCV/HIV co-infected groups

We first applied GSEA to identify functional gene sets distinct between the HIV mono- and the HCV/HIV co-infections. In total, 13 gene sets were down-regulated in the HCV/HIV co-infected group (FDR < 0.05), 12 of which were gene sets related to cell cycle check point and mitosis. The most up-regulated pathway was platelet degranulation pathway with a significant change (FDR < 0.05).

### Global gene expression profiles between HCV and HIV mono-infected groups, HCV mono- and HCV/HIV co-infected groups

**Figure 1:**
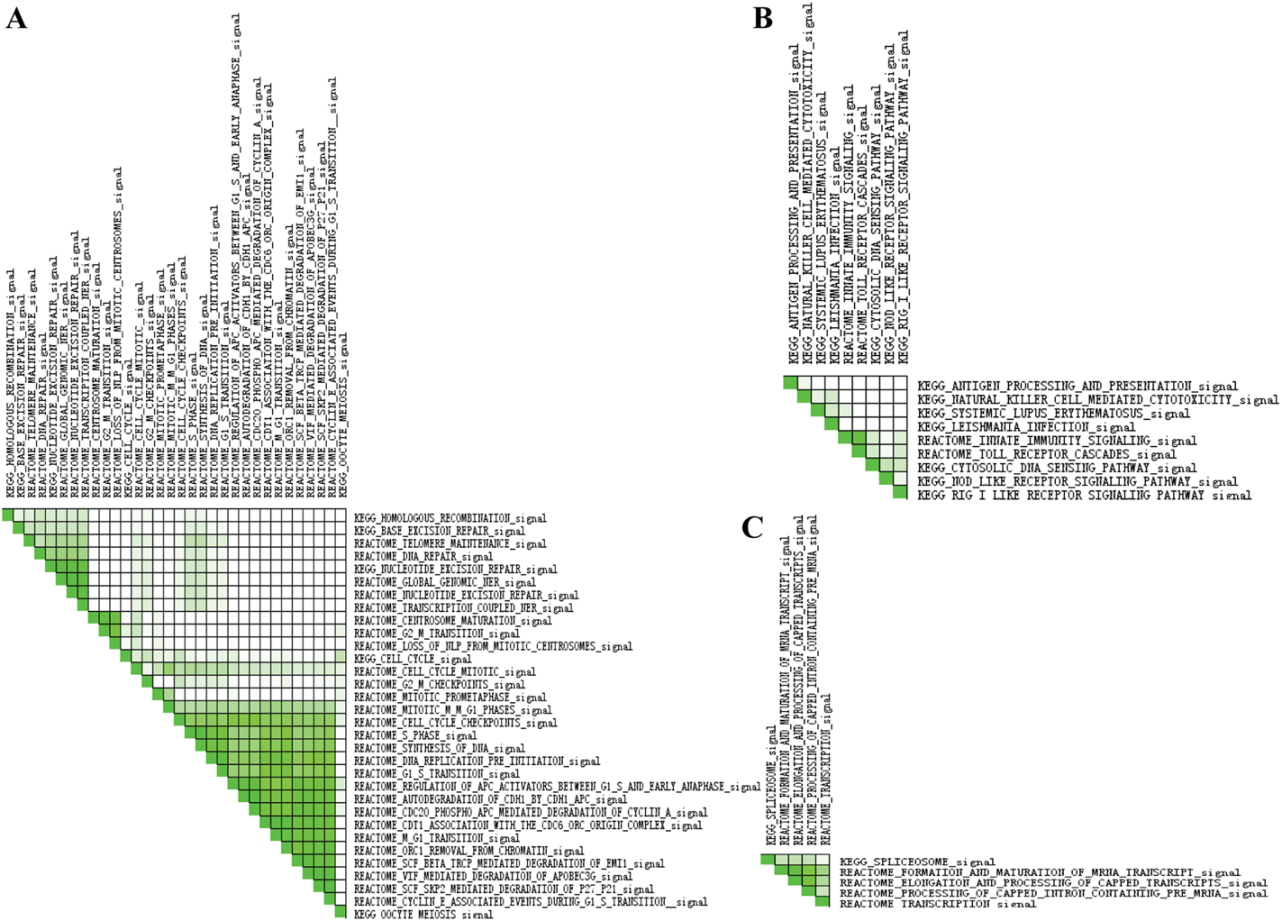
**Leading edge analyses for the gene sets related to cell cycle, immune response and transcriptional regulation identified between HIV and HCV mono-infected groups.** Gene sets related to cell cycle **(A)**, immune response **(B)** and gene expression **(C)** identified between HIV- and HCV-infected groups were analyzed by the leading edge analyses of GSEA software. The Set-to-Set maps use color intensity to show the overlap between subsets: the darker the color, the greater the overlap between the subsets.

**Figure 2:**
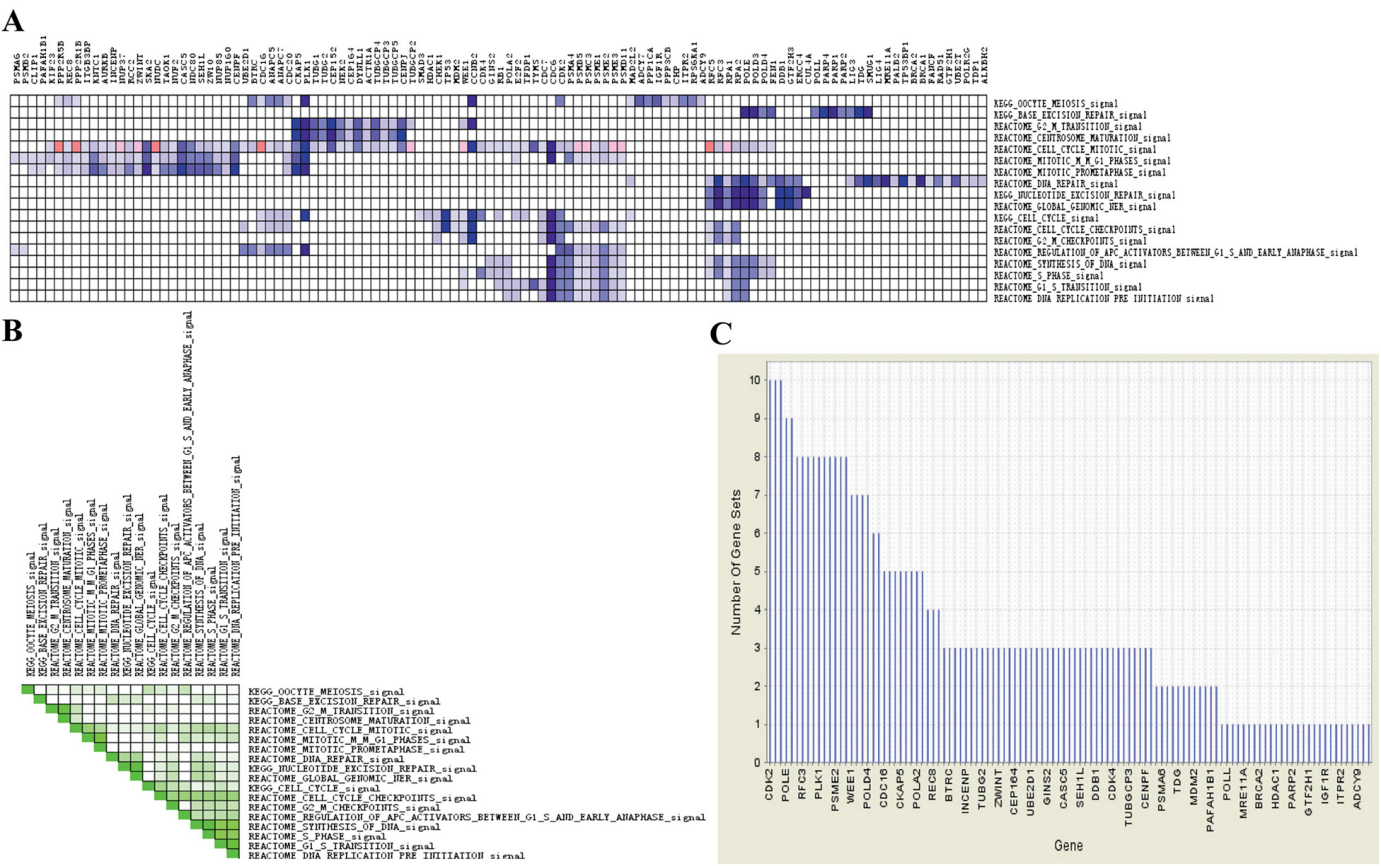
**Leading edge analyses for the gene sets related to cell cycle identified in comparison between HCV/HIV co-infected group and HCV mono-infected group.** Gene sets related to cell cycle control identified in comparison between HCV/HIV co-infected group and HCV mono-infected group were analyzed by the leading edge analyses of GSEA software. **A**. The heat map shows the genes in the leading edge subsets. In a heat map, expression values are represented as colors, where the range of colors (red, pink, light blue, dark blue) shows the range of expression values (high, moderate, low, and lowest); **B**. The Set-to-Set maps use color intensity to show the overlap between subsets: the darker the color, the greater the overlap between the subsets; **C**. graph shows each gene and the number of subsets in which it appears.

**Figure 3:**
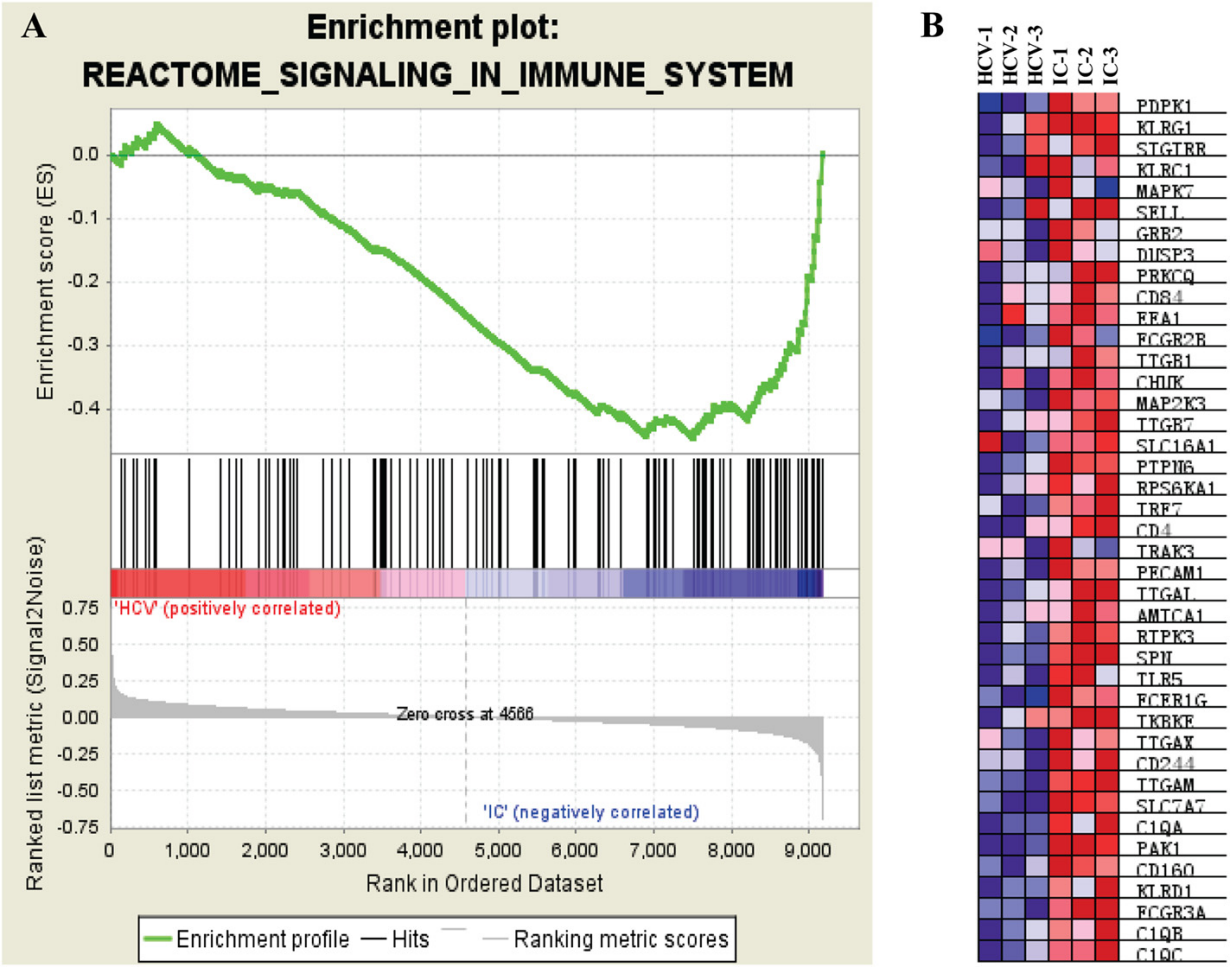
**Enrichment plot and heat map for the gene set of immune system identified in comparison between HCV/HIV co-infected group and HCV mono-infected group. A**. Enrichment plot for CD4+ T cells from comparison between HCV/HIV co-infected group and HCV mono-infected group. Bottom, plot of the ranked list of all genes. Y axis, value of the ranking metric; X axis, the rank for all genes. Genes whose expression levels are most closely associated with the HCV mono-infected group or HCV/HIV co-infected group get the highest metric scores with positive or negative sign, and are located at the left or right edge of the list. Middle, the location of genes from the gene set immune system within the ranked list. Top, the running enrichment score for the gene set as the analysis walks along the ranked list. The score at the peak of the plot is the enrichment score (ES) for this gene set and those genes appear before or at the peak are defined as core enrichment genes for this gene set. **B**. Heat map of the genes within the gene set of immune system corresponding to **A**. The genes that contribute most to the ES, i.e., genes that appear in the ranked list before or at the peak point of ES, are defined as core enrichment genes and highlighted by the red rectangle. Rows, genes; columns, samples. Range of colors (red to blue) shows the range of expression values (high to low).

We next compared the global gene expression profiles between the HCV and HIV mono-infected individuals, and between the HCV mono- and HCV/HIV co-infections. It is interesting to note that the majority of gene sets up-regulated in the HIV mono-infections also show high enrichment in the HCV/HIV co-infections when compared with the HCV mono-infections. According to their biological function, these gene sets could be mainly divided into (1) cell cycle; (2) immune response; and (3) gene expression (regulation). In the cell cycle category, 32/88 and 18/67 gene sets with an FDR < 0.05 were respectively identified to be significantly up-regulated in the HIV-infected group and in the HCV/HIV co-infected group, respectively. The leading edge analysis revealed that the majority of these genes appear to engage in G1/S and G2/M transitions (Figure 1/ Figure 2). In relation to immune response, genes involved in the innate immune response, particularly, in pathogen-associated molecular patterns (PAMPs) recognition, were shown an increased expressions in the HIV mono-infections and contributed most to the enrich score (Figure 1B). In the HCV/HIV co-infected group, the enrichment plot and heat map of the genes involved in this pathway were shown as representatives in Figure 3. Again, the most generally up-regulated gene sets identified in the HCV/HIV co-infected individuals were innate immunity signaling. These included natural killer cell mediated cytotoxicity, toll like receptor signaling pathways, NOD-like receptor signaling pathways and complement activation. While analyzing gene sets related to gene expression, a little difference existed between these two pairwise comparisons. Although both the HIV mono-infected and HCV/HIV co-infected individuals displayed up-regulation of many genes that involved in mRNA maturation (Figure 1C), genes involved in ribosome formation were significantly down-regulated in the HCV/HIV co-infections when compared with HCV mono-infections. Besides, genes involved in the metabolic pathways were also differently regulated. Gene sets including carbohydrate, lipid, amino acid, nucleotide and even vitamins metabolism were all increased in the HCV/HIV co-infected group. On the contrary, only genes function in amino acid metabolism was detected in the HIV mono-infected group when compared with the HCV mono-infected group.

### GPCR signaling pathway was up-regulated in HCV mono-infected individuals no matter compared with HIV or HCV/HIV-infected individuals

Besides the above mentioned gene sets, there still existed some other pathways that showed high overlap between these two parewise comparisons (HCV-mono versus HIV-mono, HCV/HIV co-infected versus HCV-mono). Among them, the gene sets involved in signal transduction were top listed. Contrary to the cell cycle category, these gene sets were up-regulated in HCV mono-infected individuals. Further analysis revealed that nearly these entire gene sets could be directly or indirectly associated with GPCR signaling pathway.

### Validation of differentially expressed genes

**Figure 4:**
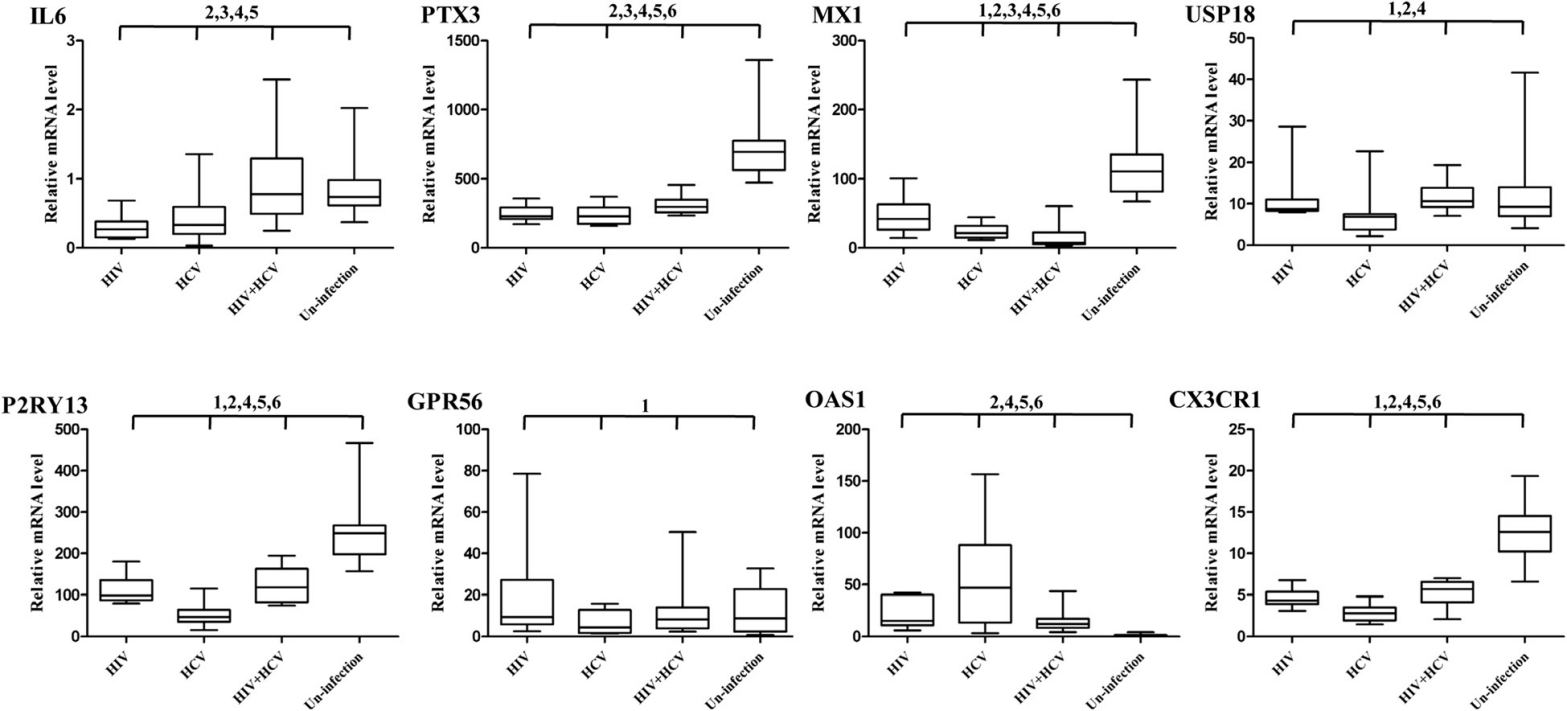
**Quantitative real time RT-PCR validations of differentially expressed genes.** The mRNA levels of selected genes were measured in HCV, HIV co-/mono-infected individuals (16 individuals in each group) by quantitative real time RT-PCR. And the relative mRNA value was calculated as described in methods. Box-plot illustrated the medians with 25% and 75%; error bars indicate 5% and 95% percentiles. 1–6 represent comparisons between HCV infection and HIV infection, HCV and HIV/HCV co-infection, HIV and HIV/HCV co-infection, HCV infection and uninfection, HIV infection and uninfection, and HIV/HCV co-infection and uninfection with *p* < 0.05, respectively.

To confirm the DE genes from the microarray analysis, the mRNA levels from each paired comparison were selected and quantitated by qRT-PCR. These genes included P2RY13, Mx1, IL6, PTX3, GPR56, OAS1, CX3CR1, USP18 (Figure 4) and FCGR3A, CCR1, TLR4, IFI44L, CD38 (Supplemental Figure 1). The RNA isolated from the CD4^+^ T cells of each individual (16 individuals in each group) was used. And the blood samples from un-infected males with marched ages were collected as control. Expression levels of these genes in four groups were all evaluated (Figure 4) and compared with the results obtained by microarray (Table 2). The qRT-PCR results showed that the expressing profiles of 6 genes (PTX3, IL6, P2RY13, OAS1, CX3CR1 and USP18) exactly matched with the observation in microarray assays; another 2 genes (Mx1 and GPR56) displayed partial similarity at least in one express pattern out of three pairwise comparisons. These results largely confirmed the data from the microarray assays. Besides, the mRNA levels of all the selected genes in un-infected persons with matched ages were also analyzed. As shown in Figure 4 and Table 2, the expression levels of CX3CR1, PTX3, MX1 and P2RY13 decreased significantly in infected groups as compared to the uninfected healthy group; while the expression level of OAS1 elevated in the infected groups. There is one gene (USP18) showing altered expressions only in HCV-infections. For IL6, lower expression levels were observed both in the HCV and HIV infections. Other immune related genes like FCGR3A and CCR1 were significantly upregulated while CD38 were decreased in co-infected group as compared to the mono infected or uninfected groups (Additional file 3: Figure 1).

**Table 2:** Quantitative real time PCR validations of differentially expressed genes

**Gene symbol**	**Method**	**HIV + HCV vs HCV**	**HIV + HCV vs HIV**	**HCV vs HIV**	**HCV vs None-infection**	**HIV vs None-infection**	**HCV + HIV vs None-infection**
**IL6**	MA	Up	Up	NS	ND	ND	ND
	qPCR	Up	Up	NS	Down	Down	NS
**PTX3**	MA	Up	Up	NS	ND	ND	ND
	qPCR	Up	Up	NS	Down	Down	Down
**Mx1**	MA	Up	NS	Down	ND	ND	ND
	qPCR	Down	Down	Down	Down	Down	Down
**USP18**	MA	Up	NS	Down	ND	ND	ND
	qPCR	Up	NS	Down	Down	NS	NS
**P2RY13**	MA	Up	NS	Down	ND	ND	ND
	qPCR	Up	NS	Down	Down	Down	Down
**GPR56**	MA	Up	NS	Down	ND	ND	ND
	qPCR	NS	NS	Down	NS	NS	NS
**OAS1**	MA	Up	NS	NS	ND	ND	ND
	qPCR	Up	NS	NS	Up	Up	Up
**CX3CR1**	MA	Up	NS	Down	ND	ND	ND
	qPCR	Up	NS	Down	Down	Down	Down

**NOTE**. VS, versus; NS, not significantly changed; ND, not detected; qPCR, quantitative real-time polymerase chain reaction; MA, microarray analysis.

## Discussion

Co-infection with HIV and HCV is common, and increasing evidence indicated that each can alter the course of infection of the other one [16]. However, little is known on the molecular basis of HIV and HCV interactions and their modulations on host responses. In this study, the gene expression profiles of CD4^+^ T cells in the treatment-naive HCV, HIV mono-infected and the HCV/HIV co-infected individuals were evaluated. For each cell subset, pairwise comparisons were performed and differentially expressed genes and significantly altered pathways were identified.

By using the GSEA analysis, groups of comprehensive pathways were identified. At first glance, it was obvious to notice that the major altered gene sets indentified in comparison between the HCV/HIV co-infections and HCV mono-infections were nearly same with those indentified in comparison between HIV and HCV mono-infections. Considering that CD4^+^ T cell was the major target of HIV and only a little specific CD4^+^ T cells was detected in the HCV infected individuals [16,17], it was reasonable that HCV only contribute a little to the global gene changes of CD4^+^ T cells in HCV/HIV co-infections (Table 1).

One significant proportion of differentially expressed genes was associated with cell cycle which was significant up-regulated in both the HIV mono- and HCV/HIV co-infected individuals as compared with HCV mono-infected individuals (Figure 2). Functional analysis revealed that most of these gene sets involved in G1/S and G2/M transitions. In relate to G1/S transition, till now only one study has reported this phenomenon and the reason why HIV prefers to drive cells out of G1 to S phase is not clear yet [18]. One possible explanation is that the G1/S transition may regulate the latency of HIV [19]. The promotion of G1/S transition may facilitate HIV integration as indicated by the up-regulation of genes engaged mainly in DNA repair. The latter has been assumed to be required for the integration of HIV [20,21]. Another most affected phase was the G2/M transition [22,23]. As shown in Figure 2, 16 out of 33 genes that associated with G2/M transition were up-regulated in the HIV infected individuals, and most of them play important roles in the maintenance of centrosome normality and integrity. The Vpr (viral protein R) of HIV was reported to induce an accumulation of multiple centrosome-like structures in human cells that lead to cell cycle arrest or delay in the G2 phase [24], thus positively support viral replication or facilitate viral spread by triggering cell death [25]. For HIV/HCV co-infection, the cell cycle arrest in G2 phase induced by HIV would lead to a depletion of CD4^+^ T cells [25], and may partially result in a less efficient in HCV clearance [26].

The second group of significantly up-regulated gene sets in both HIV mono- and HCV/HIV co-infections was genes related to innate immune response as compared with HCV mono-infections (Figure 3). In chronic infection, such as HIV, continuous ongoing innate immune responses may contribute more to disease progression rather than to limit viral replication [26]. Although the mechanisms are very complicated, functional analysis revealed that a group of overlapped genes were engaged in NF-κB regulation among these different innate immune pathways. This finding was also supported by other studies that NF-κB was mediated by Vpr and played a major role in HIV gene expression and pro-inflammatory cytokines induction [27-30].

The third group of gene sets that is usually modulate by virus is the host cell transcription apparatus [31]. By providing direct gene pattern, our results confirmed that this strategy was also exploited both in the HIV mono- and HCV/HIV co-infected individuals. As seen in Figure 1C, many of the critical components that engaged in mRNA formation, elongation and maturation were up-regulated in the HIV mono- and HCV/HIV co-infected individuals. These implied that through up-regulating the mRNA editing genes, the HIV virus could facilitate the production of maturated transcripts which were needed for its own translation.

Gene sets in metabolic pathways, including carbohydrate, lipid, fatty acid, amino acid, nucleotide and even vitamin metabolisms, were all significantly changed in the co-infected group. Both of these two viruses have shown abilities to modulate partial of these pathways to facilitate their infections [32-34]. The abnormalities in the whole metabolic net in co-infections may be a result of HIV-HCV interaction. By complement to each other, the damaging effects would be definitely aggravated, thus lead a more rapid disease progression in co-infections.

Results from our study also detected several pathways up-regulated in HCV infections. All of these pathways were directly or indirectly relate to pathway of GPCR signaling (Figure 1). By modulation several major effectors such as adenylyl cyclase, phospholipase C and the mitogen activated protein kinases (MAPKs), GPCR are involved in many diverse signaling events including visual sense, smell, immune system regulation and inflammation [35,36]. The significance of HCV on modulating GPCR signaling is not well understood yet. We speculate that the activation of GPCR signaling as exemplified by upregulation of chemokines (e.g., CCL22) may play important roles in lymphocytes chemotaxis, which could promote the activated lymphocytes to inflammatory sites and facilitate viral clearance [37]. However, an inappropriate activation of chemotaxis may also lead to unexpected damage of un-infected cells and accelerate the disease progression. Activation of GPCR pathways would lead to an increase in amount and duration of intracellular cyclic adenosine 3', 5’-monophosphate (cAMP) levels [38]. As an important regulator of immune cells, cAMP has a dual and opposite role during HIV infection [39]. cAMP can limit viral entry and replication [40,41], while it may also reduce HIV-specific antiviral immune responses and promote T cell dysfunctions [42,43]. The exact role of HCV-activated GPCR signaling on CD4^+^ T cells function and HIV infection needed to be further determined.

## Conclusion

This study for the first time analyzed the impact of HIV/HCV co-infection at gene transcription level. By comparing with HCV and HCV/HIV co-infection, HIV infection displayed the great impact on gene expression profile of CD4^+^ T cells. Genes related to cell cycle arrest and T cell dysfunction were significantly mediated in HIV and HIV/HCV infected individuals which may practically explain the acceleration of HCV related disease progression by HIV infection and control of HIV replication might improve HCV specific CD4^+^ T-cell response in the HCV/HIV co-infected individuals.

## Methods

### Treatment-naive HCV/HIV mono-/co-infected individuals

A male population of Chinese was recruited from an ongoing voluntary-based HIV/AIDS surveillance study in Shenzhen, from September 2009 to December 2010. Written content was obtained from the participants. Ethics approval was obtained from The Joint Chinese University of Hong Kong–New Territories East Cluster Clinical Research Ethics Committee. All participants have been screened for HIV (Beijing Wantai Biological Pharmacy Enterprise CO., LTD, Beijing), HCV (Beijing Wantai Biological Pharmacy Enterprise CO., LTD, Beijing) and HBV (Beijing Wantai Biological Pharmacy Enterprise CO., LTD, Beijing) antigens and antibodies by standard ELISA analysis recommended by The Center of Disease Control and Prevention of China (guidelines of CDC, China). HIV infection was further confirmed by western blot (HIV Blot 2.2 WB; Genelabs Diagnostics, Singapore) following the National Guideline for Detection of HIV/AIDS of China. In order to rule out the confounding factors including treatment responses and drug resistant bias, all HIV + samples were tested for CD4 counts with flow cytometry, and drug resistant mutations by genotyping analysis. All enrolled HIV + individuals are antiretroviral-naïve with CD4 counts ≥300 cells/μl and without baseline drug resistant mutation. Based on the stringent criteria described above, 24 samples from each infected group (HIV mono-infected, HCV mono-infected and HIV/HCV co-infected group) were selected in this study. For microarray analysis, each group contained 3 biological replicates with 8 individuals in each replicates. To validate our findings by quantitative real-time PCR (qRT-PCR), 24 healthy individuals were further enrolled. Their demographic and epidemiological data have been documented in Additional file 4: Table S1. The average age was similar in all the study groups (29.5, 33.0, 31.4 and 27.5 years for HIV mono-infection group, HCV mono-infection group, HIV/HCV co-infection group and non-infected group, respectively). The CD4 counts were 433 ± 101, 839 ± 234, 524 ± 219 and 651 ± 210 (mean ± SD, cells/μl) for HIV infected, HCV infected, HIV/HCV co-infected and un-infected group, respectively.

### Purification of CD4^+^ T cells and RNA isolation

Peripheral blood mononuclear cells (PBMCs) were separated and purified immediately after obtaining blood samples (30 ml whole blood from each individual) by Ficoll-Hypaque separation. Fresh CD4^+^ T cells were then obtained by positive isolation with use of microbead immunoselection (Miltenyi Biotec, Oslo, Norway). To maximize the RNA yields and minimize possible variations in gene expression profiles, the experiments were strictly followed as described in a previous study [44]. The purity of isolated CD4^+^ T cells was measured by Flow-cytometric analysis of cell markers (CD4). And it demonstrated that 98.1% ± 0.013 (mean ± SD), 98.5% ± 0.072, 97.6% ± 0.021 and 98.5% ± 0.012 of purified CD4^+^ cells were single positive for the CD4 marker in HIV infected, HCV infected, HIV/HCV co-infected and un-infected group, respectively. For microarray analysis, RNA pooling method was used to reduce the noise and enhance reliability when many subjects were pooled without loss of individual specific information [45]. In each infection group, samples were divided into 3 subgroups for independent replicates. In each subgroup, equal amount of CD4^+^ T cells from 8 individuals were pooled together for RNA isolation and the followed gene expression profile studies. The left CD4^+^ T cells were stored at -80°C freezer for validation. RNA was isolated using RNAeasy Total RNA Isolation Kit (Qiagen, Germany) and applied for microarray assays.

### Microarray assays

Microarray assays were performed by using Affymetrix GeneChip Human Gene 1.0 ST Array (Affymetrix, Santa Clara, CA). A cRNA kit (The Affymetrix GeneChip Whole Transcript Sense Target Labelling Assay) was used for complementary RNA (cRNA) generation, hybridization, and array processing. The images were acquired by the Affymetrix Scanner 3000 7G Plus and the CEL files were imported into the program Partek Genomic Suite (version 6.4, Partek Inc, St Louis, MO) and the robust multi-chip average (RMA) was normalized. Two-way ANOVA test was applied to identify differentially expressed (DE) genes (fold change >2 and adjust *p* < 0.05).

### Gene set enrichment analysis

To further understand the biological meanings, Gene set enrichment analysis (GSEA) analysis (version 2.0, Broad Institute http://www.broad.mit.edu/gsea) was used. First, a ranked list was obtained by ranking all genes according to the correlation between their expression levels and the group distinctions using the metric signal to noise ratio. Then the association between a given gene set and the group was measured by the non-parametric running sum statistic termed the enrichment score (ES). To estimate the statistical significance of the ES, a nominal *p* value was calculated by permuting the genes 1,000 times. To adjust for multiple hypotheses testing, the maximum ES was normalized to account for the gene set size (NES) and the false discovery rate (FDR). The gene sets used were from Molecular Signatures Database (MsigDB), catalog C2 (version 3.0) functional sets, subcatalog canonical pathways, which include 880 gene sets from pathway databases. These gene sets were collected from online databases such as Bio-Carta, React, and KEGG (Kyoto Encyclopedia of Genes and Genomes).

### Quantitative real-time RT-PCR (qRT-PCR)

RNA isolated from the CD4^+^ T cells of each participant was reverse-transcribed into cDNA using Reverse Transcription System (Promega, USA). qRT-PCR was carried out by using ABI 7500 Real-Time PCR system with Power SYBR Green Master Mix (Applied Biosystems, USA). Primer sequences are listed in Additional file 5: Table S4. The relative mRNA level of each gene was calculated as following formula: R = 2^CT (GAPDH-X)^ x10^3^, x represent CT value of each gene. Each RT-PCR experiment was performed three times.

### Statistical analysis

Data were analyzed with SPSS version 13.0 (SPSS Inc., Chicago, IL, USA). Nonparametric test was used for parewise comparisons. *P* value < 0.05 was considered as statistically significant.

## Competing interests

The authors have declared that no competing interests exist.

## Authors’ contributions

MLH, ZRY, HK and JZ designed the research. JZ, LY, JL, YC, LC, JC, YS and ZL carried out all the experiments. LY and JL analyzed the data. LY wrote the manuscript. And all authors reviewed the manuscript.

## Supplementary Material

Additional file 1: Table S2 Differently expressed transcript identifiers in each comparison. Click here for file

Additional file 2: Table S3 Pathways identified by gene set enrichment analysis (FDR < 0.05). Click here for file

Additional file 3: Figure S1 Quantitative real time RT-PCR validations of differentially expressed genes. Click here for file

Additional file 4: Table S1 Participant cohort. Click here for file

Additional file 5: Table S4 Primers for Quantitative Real-time PCR Analysis. Click here for file
