# Supplementary material for: Gene expression profiling of CD4+ T cells in treatment-naive HIV, HCV mono- or co-infected Chinese
Source: Virol J. 2014 Feb 13;11:27. doi: 10.1186/1743-422X-11-27 (PMC3943807; doi:10.1186/1743-422X-11-27)
Supplement: Additional file 3: Figure S1 — Quantitative real time RT-PCR validations of differentially expressed genes. [file 1743-422X-11-27-S3.docx]

**

**

**Figure S1. Quantitative real time RT-PCR validations of differentially expressed genes.** The mRNA levels of selected genes were measured in HCV, HIV co-/mono-infected individuals (16 individuals in each group) by quantitative real time RT-PCR. And the relative mRNA value was calculated as described in methods. Box-plot illustrated the medians with 25% and 75%; error bars indicate 5% and 95% percentiles. 1–6 represent comparisons between HCV infection and HIV infection, HCV and HIV/HCV co-infection, HIV and HIV/HCV co-infection, HCV infection and uninfection, HIV infection and uninfection, and HIV/HCV co-infection and uninfection with *p* < 0.05, respectively.
