# Supplementary material for: Gene expression profiling of CD4+ T cells in treatment-naive HIV, HCV mono- or co-infected Chinese
Source: Virol J. 2014 Feb 13;11:27. doi: 10.1186/1743-422X-11-27 (PMC3943807; doi:10.1186/1743-422X-11-27)
Supplement: Additional file 5: Table S4 — Primers for Quantitative Real-time PCR Analysis. [file 1743-422X-11-27-S5.doc]

**Table S4.** Primers for Quantitative Real-time PCR Analysis

| **Gene** | **Accession No.** | **Sequences of primers** |
| --- | --- | --- |
| IL6 | NM_000600 | F 5’-AGAGTAGTGAGGAACAAG-3’  R 5’-GCAGAATGAGATGAGTTG-3’ |
| PTX3 | NM_002852 | F 5’-GCTACCACTGTTGAGATG-3’  R 5’-CCAGAGAAGGCTAATGTT-3’ |
| Mx1 | NM_002462 | F 5’-AGGACTACGAGATTGAGAT-3’  R 5’-TTATGCCAGGAAGGTCTA-3’ |
| USP18 | NM_017414 | F 5’-AATCTGTCAGTCCATCCT-3’  R 5’-GTTGCTGTCTTCTTCCTT-3’ |
| P2RY13 | NM_176894 | F 5’-TGACTGCCGCCATAAGAA-3’  R 5’-CTGTGGTGTTCATTGCTTCC-3’ |
| GPR56 | NM_201524 | F 5’-GTGAGACCGTCAGGAGAG-3’  R 5’-CACCGAGGAGACCATCAG-3’ |
| OAS1 | NM_016816 | F 5’-TCCACCTGCTTCACAGAACTACA-3’  R 5’-TGGGCTGTGTTGAAATGTGTTT-3’ |
| CX3CR1 | NM_001337 | F 5’-TGTGTTCCTGTCCATATTCTACT-3’  R 5’-GCTTCTTGCTGTTGGTGAG-3’ |
| GAPDH | NM_002046 | F 5’-GATTCCACCCATGGCAAATTCCA-3’  R 5’-TGGTGATGGGATTTCCATTGATGA-3’ |
